# Draft Genome Sequence of *Burkholderia dolosa* PC543 Isolated from Cystic Fibrosis Airways

**DOI:** 10.1128/genomeA.00043-14

**Published:** 2014-02-13

**Authors:** Matthew L. Workentine, Michael G. Surette, Steve P. Bernier

**Affiliations:** a Farncombe Family Digestive Health Research Institute, Faculty of Health Sciences, Department of Medicine, McMaster University, Hamilton, Ontario, Canada; b Department of Biochemistry and Biomedical Sciences, Faculty of Health Sciences, McMaster University, Hamilton, Ontario, Canada

## Abstract

*Burkholderia dolosa* is a member of the *Burkholderia cepacia* complex, a group of opportunistic bacterial pathogens often associated with fatal chronic infections in the lungs of patients suffering from cystic fibrosis (CF). Here, we announce the draft genome sequence of *B. dolosa* PC543 (LMG 19468), a CF airway isolate.

## GENOME ANNOUNCEMENT

The *Burkholderia cepacia* complex (BCC) is a versatile group of closely related bacteria (1) currently divided into 18 species (2, 3). The BCC have the potential to cause chronic and severe airway infections in persons with cystic fibrosis (CF) (2). Although the prevalence of BCC species in CF airways is most commonly from *Burkholderia cenocepacia* and *Burkholderia multivorans* (2), other BCC species, like *Burkholderia dolosa*, have caused outbreaks (4, 5).

Here, we report the genome sequence of *B. dolosa* strain PC543, an isolate obtained from the airway of a CF patient (6), which was also named LMG 19468 since its deposition into the LMG Bacteria Collection. While a fully annotated genome of *B. dolosa* strain AU0158 is already present in the *Burkholderia* Genome Database (7) and part of the *B. dolosa* sequencing project (http://www.broadinstitute.org/annotation/genome/burkholderia_dolosa), the current sequence contains several regions with missing nucleotides (rich in Ns from the scaffold assembly) that we randomly identified by mapping transposon insertions from a genetic screen made in *B. dolosa* PC543. Therefore, to correctly map our transposon insertions and to get the missing parts from *B. dolosa* AU0158, we sequenced the genome of one our transposon candidates.

Genome sequencing was performed using Illumina HiSeq sequencing technology. Briefly, genomic DNA was extracted using the Wizard Genomic DNA purification kit (Promega, Madison, WI), according to the manufacturer’s instructions. The library was prepared using the Nextera XT kit (Illumina) and sequenced to a coverage depth of 80×. Contigs were assembled using the A5 Pipeline (8), which performs automated quality filtering and error correction on the reads prior to assembly. The pipeline uses ibidem A (9) to assemble the reads, followed by contig scaffolding with SSPACE (10). The contigs were ordered by whole-genome alignment to the sequenced *B. dolosa* AU0158 genome using Mauve (11). Several misassemblies were identified and corrected manually, yielding a total of 64 contigs with an N_50_ of 196,616 and a maximum contig size of 411,869. The contigs were submitted to NCBI for annotation using the Prokaryotic Genome Annotation Pipeline 2.0.

The current sequence of *B. dolosa* PC543 harbors a transposon insertion (12) in gene BDSB_15450. Similar to the reference *B. dolosa* AU0158 sequence, the genome of strain PC543 has three chromosomes with predicted sizes of 3.31, 2.16, and 0.82 Mb, respectively, for a total genome size of 6.3 Mb. The genome has a G+C content of 67.1%, with a predicted 5,874 genes (open reading frames [ORFs]) and 5,739 proteins (protein coding sequences [CDS]).

### Nucleotide sequence accession numbers.

The *B. dolosa* PC543 whole-genome shotgun project has been deposited at DDBJ/EMBL/GenBank under the accession no. AWRY00000000. The version described in this paper is the first version, AWRY01000000.
